# The Diagnostic Value of Cell Block Immunohistochemistry Combined with Imaging For Difficult Pleural Effusion

**DOI:** 10.2174/0115734056446228260706113151

**Published:** 2026-07-21

**Authors:** Hui Yu, Zhi Yong Shen

**Affiliations:** 1 Translational Medicine Center, Nantong Tumor Research Institute; Affiliated Tumor Hospital of Nantong University, Nantong Tumor Hospital, No. 30, North Tong-yang Road, Pingchao Town, Tongzhou District, Nantong, Jiangsu 226361, China

**Keywords:** Pleural effusion, Non-pulmonary origin, Liquid-based cytology, Cell block, Immunohistochemistry, CT, PET-CT

## Abstract

**Introduction/ Background:**

Advanced pulmonary carcinoma frequently metastasises to the chest cavity, resulting in malignant pleural effusion (PE). Consequently, when PE is of pulmonary origin, diagnosis is generally straightforward. However, when PE originates from non-pulmonary sites, diagnosis becomes more challenging. This study aims to evaluate the diagnostic utility of combining cell block immunohistochemistry (IHC) with imaging modalities in diagnostically challenging cases of PE.

**Materials and Methods:**

We collected PE data from difficult-to-diagnose cases admitted to Nantong Tumor Hospital from January to December 2024. Malignant PE of non-pulmonary origin and benign PE were defined as difficult cases. For each patient, pleural fluid underwent liquid-based cytology, cell block IHC, and imaging to differentiate between benign and malignant etiologies.

**Results:**

Among the 23 patients included, 9 presented with benign PE and 14 with malignant PE.

1. Benign pleural fluid cases: IHC analysis of cell blocks revealed proliferating mesothelial cells. CT identified six cases of PE, including five with concomitant pneumonia. PET-CT was performed in three patients; results indicated pneumonia in two cases and no significant abnormal uptake in the remaining case.

2. Malignant PE cases: The primary origins were the digestive system (eight cases), breast (five cases), and gynecological system (one case).

3. Cases of gastrointestinal origin: One patient was diagnosed with a gallbladder tumor *via* PET-CT; one with a pancreatic tail tumor *via* CT; two with colon malignancy *via* CT; and one with gastric cancer *via* CT. The remaining three cases had their primary lesions surgically removed, and CT revealed abdominal metastases.

4. Cases of breast origin (five cases): CT imaging showed lung metastasis in three cases, liver metastasis in two cases, and bone metastasis in two cases.

5. Case of gynecological origin: CT showed PE and lung metastasis.

**Discussion:**

IHC can help determine the origin of PE. However, IHC sometimes provides only a general direction, such as suggesting that malignant cells arise in the digestive tract. The digestive tract includes a wide range of organs (*e.g*., gastrointestinal tract, liver, gallbladder, pancreas). Therefore, to accurately localize the source of PE to specific organs or tissues, imaging examination is indispensable.

**Conclusion:**

Cell block IHC is effective for determining the origin of PE, while imaging is useful for localization and identification of metastatic lesions. Combining these modalities helps differentiate between benign and malignant etiologies in difficult-to-diagnose PE cases.

## 1 INTRODUCTION

Malignant pleural effusion (PE) is a serious complication of advanced tumors and significantly affects both quality of life and patient prognosis [1]. Although malignant PE is most commonly caused by lung cancer [2, 3], it may also originate from metastatic tumors of non-pulmonary origin, such as breast cancer [4], gastrointestinal cancer [5], esophageal cancer [6], ovarian cancer [7], or lymphoma [8].

Accurate identification of the primary source, particularly in cases of non-pulmonary origin, is of great significance for determining tumor staging, searching for the primary lesion, and developing personalised treatment plans. Furthermore, a common and challenging clinical scenario involves patients with a known history of malignancy who develop new PE, which is initially suspected to result from tumor metastasis. However, some of these cases are ultimately confirmed to have benign PE (*e.g*., due to infection, inflammation, *etc*. [9]). Misdiagnosis as malignant PE may lead to unnecessary anti-tumor treatment and impose additional physical and psychological burdens on patients. Therefore, accurate diagnosis is crucial in difficult cases of both malignant PE from non-pulmonary sources and benign PE in patients with a history of tumors.

Liquid-based cytology (LBC) examination of PE is the preferred straightforward method for diagnosing malignant PE [10]. However, its sensitivity has significant limitations, especially in cases of low tumor cellularity, poor differentiation, or atypical morphology, leading to a high rate of misdiagnosis. Reactive proliferation of mesothelial cells [11] can be easily confused morphologically with malignant mesothelioma or metastatic adenocarcinoma cells [12], which is one of the main reasons why benign PE in patients with a history of tumors is misdiagnosed as malignant PE.

Imaging examinations such as chest computed tomography (CT) [13] and positron emission tomography-computed tomography (PET-CT) [14] are indispensable for evaluating pleural lesions and identifying primary and metastatic lesions. However, their specificity is insufficient, and benign diseases (*e.g*., tuberculous pleurisy and chronic inflammatory changes) can also exhibit imaging manifestations resembling malignant ones, such as pleural thickening [15] and tumors [16]. This makes it difficult to diagnose the nature of PE or reliably rule out metastasis based solely on imaging.

The integration of artificial intelligence (AI) into medical imaging has garnered significant research attention in recent years [17]. Specifically, machine learning-based models can provide valuable information for the differential diagnosis of PE [18]. These AI-driven tools can enable clinicians to diagnose and treat PE more effectively [19].

The application of a series of specific antibody markers *via* immunohistochemistry (IHC) [20] on PE cell blocks has effectively improved the detection rate of malignant cells [21]. Furthermore, IHC can accurately identify tumor types and their cellular origins (*e.g*., lung adenocarcinoma [22], breast cancer [23], ovarian cancer [24], mesothelioma [25], lymphoma [26]), thereby serving as a critical tool in the diagnosis of difficult-to-diagnose PE.

Currently, few reports exist on the diagnosis of difficult PE using a combination of cytological IHC and imaging modalities.

The aim of this study is to explore the clinical utility of IHC combined with imaging in the diagnosis of non-pulmonary malignant PE and the differentiation of benign PE in patients with a history of malignant tumors or unexplained chest tightness and shortness of breath presenting at our specialized oncology center. The study workflow is outlined in Fig. (**1**). The objective is to provide a foundation for optimizing diagnostic workflows and enhancing diagnostic accuracy for such challenging PE cases.

## 2 MATERIALS AND METHODS

### 2.1 Clinical Data

Data were collected from all patients hospitalised at Nantong Tumor Hospital from January through December 2024. A total of 121 cases of PE were examined by IHC during the study period. Imaging studies for 29 cases were performed at external hospitals. Recent imaging reports (obtained within one week) from institutions of equivalent or higher accreditation are accepted by our hospital, obviating the need for repeat imaging. IHC confirmed malignancy in 69 PE cases, identifying them as primary lung tumors.

After excluding these two categories, 23 cases remained (Fig. **2**: Flowchart).

We classified PE into straightforward and difficult cases. Pulmonary-origin PE was considered straightforward, whereas PE of non-pulmonary origin and benign PE were classified as difficult.

Pulmonary-origin PE is typically caused by late-stage lung carcinoma that has metastasised to the chest cavity, making clinical diagnosis relatively easy. In contrast, when PE is not of pulmonary origin, oncologists must perform further examinations, such as imaging studies, to identify the primary tumor source. Consequently, the diagnostic process is time-consuming, labour-intensive, and expensive, rendering these cases difficult.

In addition, we classified benign PE as challenging for the following reasons. All patients with a history of malignant tumors whose PE was diagnosed as benign *via* IHC in this cohort were required to undergo IHC examination at least twice to confirm the absence of cancer cells. Typically, when malignant cells cannot be found in the PE, patients do not receive intrapleural infusion chemotherapy; instead, they are treated with anti-inflammatory therapy or thoracic drainage. Pathologists at our institution exercise great caution when diagnosing PE as benign in patients with a history of malignant tumors. Therefore, benign PE can be considered challenging, at least to some extent.

A total of 23 patients with difficult PE were included, comprising 7 males and 16 females. Patients ranged in age from 39 to 89 years, with a mean age of 68 years. Previous medical histories included breast cancer (n=6), esophageal cancer (n=4), gastric cancer (n=4), colon cancer (n=4), thyroid cancer (n=1), and cervical cancer (n=1); the remaining 3 patients had no history of tumors (Table **1**).

Inclusion criteria were: (1) benign PE, and (2) malignant PE of non-pulmonary origin, both with complete imaging studies and pathological data. Exclusion criteria included: (1) malignant PE of pulmonary origin; (2) imaging examinations performed at other institutions; (3) incomplete imaging or pathological data; and (4) lack of clinical or pathological data.

This research was approved by the Ethics Committee of Nantong Tumor Hospital under approval number 2025-040-029. All procedures involving human participants were in accordance with the ethical standards of the relevant institutional and national committees, as well as the Declaration of Helsinki (1975, revised in 2013). Written informed consent was obtained from all patients or their legal representatives.

### 2.2 Liquid-based Cytopathology Test (LCT)

LCT was performed following the standard protocol for non-gynecological liquid-based cytology. Fresh pleural fluid specimens were transferred into 50 mL centrifuge tubes and centrifuged at 1200 rpm for 10 minutes. The supernatant was discarded, and the resulting cell pellet was resuspended in cell preservation solution prior to fixation. The fixed cell suspension was then transferred into 10 mL centrifuge tubes and centrifuged again at 1200 rpm for 10 minutes, after which the supernatant was decanted. Subsequently, buffer was added at 2-5 times the pellet volume, and the mixture was gently pipetted 2-3 times to resuspend the cells. Finally, 2-5 drops of the cell suspension were applied to glass slides and stained using routine Papanicolaou staining.

### 2.3 PE Embedding

PE specimens were centrifuged at 800 rpm for 10 minutes. The precipitate was collected and fixed with 95% ethanol for 30 minutes, followed by a second centrifugation at 800 rpm for 10 minutes. The resulting precipitate was then wrapped in embedding paper, fixed, dehydrated, infiltrated with paraffin wax, and embedded to form paraffin cell blocks. Routine sectioning and staining were subsequently performed.

### 2.4 IHC

The antibodies TTF-1, CK7, CK5/6, p63, calretinin, WT-1, and others were purchased from Fuzhou Maixin Biotechnology Development Company. Cell wax blocks were sliced into 3-4 μm sections, then baked, deparaffinized, and hydrated. EDTA high-pressure heat-induced epitope retrieval (HIER) was performed, followed by cooling and washing. Endogenous peroxidase activity was blocked with 3% hydrogen peroxide in methanol for 10 minutes. Primary antibodies were added dropwise, and the slides were incubated overnight at 4°C. After overnight incubation, the slides were brought to room temperature, washed, and incubated with the secondary antibody at 37°C for 30 minutes. The sections were then washed, counterstained with hematoxylin, differentiated, blued, dehydrated with ethanol, and mounted with a coverslip.

Positive staining was defined as brown-yellow or brown coloration observed under light microscopy.

Different antibody combinations can be used to determine the origin of tumors. Lung adenocarcinoma [27, 28] is the most common cause of PE; accordingly, Napsin A and TTF-1 [29] are the most frequently used biomarkers for this condition. Calretinin and WT-1 [30] serve as mesothelial markers to differentiate malignant from benign cells in serous fluids. GATA-3 and GCDFP-15 [31] are sensitive markers for identifying breast-origin metastatic adenocarcinoma, which is the most common malignancy among women [32].

CDX2 is routinely used to identify tumors of gastrointestinal origin [33, 34].

Table **2** lists the commonly used markers for determining the origin and tissue source of cancer cells in pleural fluid cell blocks.

### 2.5 Computed Tomography (CT)

Multiphasic CT was performed using a 64-section multidetector CT scanner (Somatom Sensation 64; Siemens Medical Systems, Erlangen, Germany) with the following parameters: tube voltage of 120 kV, tube current of 60-90 mA, slice thickness and reconstruction interval of 5 mm, pitch of 1.0, matrix of 512 × 512, and collimation of 1.0 mm. A scan acquired during a single breath-hold was performed from the lung apex to the lower edge of the pubic symphysis.

Contrast-enhanced scanning was performed using iodixanol (300 mg I/mL) or ioversol (300 mg I/mL), administered *via* an antecubital vein at a rate of 2.5-3.0 mL/s with a dose of 1.5 mL/kg body weight. The arterial phase was acquired 25-30 seconds after contrast injection, and the venous phase was acquired 51-60 seconds after injection.

Raw data were obtained with a slice thickness and interval of 1.0 mm, reconstructed, and then uploaded to a workstation for multiplanar reconstruction (MPR).

### 2.6 CT Manifestations

Benign PE: CT features include uniform water attenuation, with fluid distribution appearing as free-flowing, loculated, or fissural. Pleural manifestations include smooth, uniformly thickened pleura without nodules.

Malignant PE: CT features include pulmonary tumors, interstitial pleural involvement, focal nodular pleural thickening and enhancement, pleural thickness greater than 10 mm [35], PE accompanied by segmental atelectasis, supraclavicular and mediastinal lymphadenopathy, and multiple lung metastases.

Abdominal CT findings of malignant tumors: These include pancreatic or gastrointestinal tumors, intrahepatic metastases, multiple enlarged lymph nodes in the retroperitoneum and mesentery, and omental and mesenteric thickening suggestive of metastasis.

### 2.7 Positron Emission Tomography (PET/CT)

Four patients were examined using ^18^F-fluorodeoxyglucose positron emission tomography/low-dose computed tomography (^18^F-FDG PET/CT) (Philips, Amsterdam, The Netherlands). The CT scanning parameters were as follows: tube voltage, 140 kV; tube current, 90 mA; rotation time, 0.8 s; slice thickness, 5 mm; bed speed, 22.5 mm/s; matrix, 512 × 512; and fusion matrix, 128 × 128. PET parameters for the 2D mode were as follows: matrix size, 128 × 128; 2-6 acquisition windows; and acquisition time of 3-5 minutes per window [36].

Positive pleural FDG uptake is an accurate method for identifying malignant PE [37].

This study implemented an integrated diagnostic protocol for all patients. First, liquid-based cytology and cell block preparation were performed on PE samples. Subsequently, antibody combinations were selected for IHC staining based on the patient's medical history and cytology results. Second, patients underwent contrast-enhanced CT or PET-CT imaging. Finally, integrating IHC results (primary tumor source) with imaging findings (anatomical location and metastatic lesions) yielded a comprehensive diagnosis to guide clinical management.

### 2.8 Diagnostic standards

Diagnostic criteria for malignant PE:

IHC: Carcinoma cells are identified, and their origin is determined.

Imaging: Lung tumor nodules and pleural thickening (>10 mm) with enhancement [35] or positive FDG-PET pleural uptake [37].

Diagnostic criteria for benign PE:

IHC: No carcinoma cells are detected; only mesothelial cells are present.

Imaging: Only pneumonia and PE, with no evidence of pleural thickening or enhancement [35].

Clinical diagnostic criteria:

If both ICH and imaging indicate a benign condition, the case is classified as benign PE; If either modality indicates malignancy, the diagnosis is established as malignant PE.

## 3 RESULT

There were 9 cases of benign PE and 14 cases of malignant PE.

### 3.1 LCT Result

In benign PE, 100% (9/9) of cases exhibited atypical cells. In malignant PE, 28.6% (4/14) contained cancer cells, while 71.4% (10/14) contained atypical cells (Table **3**).

### 3.2 IHC Result

Benign effusion: In all 9 cases, a small number of atypical cells were identified as proliferating mesothelial cells by IHC. The IHC profile was as follows: HBME-1 (+), calretinin (CR) (+), WT-1 (+), TTF-1 (-), Napsin A (-), P40 (-), GATA-3 (-).

Malignant effusion: Malignancy was determined by the identification of cancer cells.

#### 3.2.1 Digestive System Origin (8/14 Cases)

The IHC profile showed EpCAM (+), WT-1 (-), CR (-), CK20 (+), CK19 (+), CDX-2 (positive in most cases), Napsin A (-), CK7 (+), TTF-1 (-). In individual cases, CDX-2 (+), CEA (+), Villin (+), SATB2 (+), and Pax-8 (-) were also observed.

#### 3.2.2 Breast Origin (5/14 Cases)

The IHC profile showed HBME-1 (-), Ep-CAM (+), WT-1 (-), calretinin (CR) (-), TTF-1 (-), Napsin A (-), GCDFP15 (+), GATA-3 (+), ER (<1%+), PR (<1%+), C-erbB-2 (2+), and mammaglobin (partially +).

#### 3.2.3 Female Reproductive Tract Origin (1/14 Case)

The IHC profile showed HBME-1 (-), EpCAM (+), calretinin (CR) (-), WT-1 (-), ER (0), PR (0), P53 (0), p16INK4a (partially positive), Ki67 (+), Pax-8 (+), and Napsin A (-) (Table **3**).

### 3.3 Imaging Results

#### 3.3.1 Benign PE (9 Cases)

PET-CT was performed in 3 cases (2 showed pneumonia; 1 showed no significant abnormal uptake). CT identified the remaining six PE cases, five of which had concomitant pneumonia.

#### 3.3.2 Malignant PE (14 Cases)

Among the 8 cases of gastrointestinal origin identified by IHC, imaging confirmed PE in all eight. PET-CT identified a gallbladder tumor in one patient. CT revealed a pancreatic tail tumor in one patient, colon malignancies in two patients, and gastric cancer in one patient. In the remaining three cases, the primary lesions had been surgically resected, and CT disclosed metastatic lesions or enlarged abdominal lymph nodes.

#### 3.3.3 Breast Origin (5 Cases)

CT confirmed PE in all five cases. CT revealed lung metastases in 3 patients, liver metastases in 2 patients, and bone metastases in 2 patients.

#### 3.3.4 Gynecological Origin (1 Case)

CT revealed PE with lung metastasis (Table **3**).

### 3.4 Treatment Methods

#### 3.4.1 Benign PE

Symptomatic treatment for benign PE mainly included PE drainage, anti-inflammatory therapy, and fluid replacement.

In one patient, the possibility of PE caused by tuberculous pleurisy or connective tissue disease could not be excluded. Therefore, she was referred to another specialized hospital for further diagnosis and treatment, which she ultimately requested.

#### 3.4.2 Malignant PE

The management of malignant PE encompassed three primary aspects:

1. **Pleural fluid management:** pleural drainage and intrapleural instillation of agents (*e.g*., cisplatin, fluorouracil, recombinant human endostatin, and cetriptyline).
2. **Systemic antitumor therapy:** *e.g*., trastuzumab for breast-origin tumors, bevacizumab and trastuzumab for gastric cancer, and oral capecitabine for pancreatic tail lesions.
3. **Supportive care:** oxygen therapy, nutritional support, and pain relief (Table **4**).

## 4 DISCUSSION

Pleural fluid is one of the most common specimens encountered in cytology laboratories and plays a pivotal role in both clinical diagnosis and molecular testing. Fluid aspiration is a therapeutic intervention that alleviates symptoms and is often the only available specimen for patients with advanced-stage disease [38].

Metastatic tumors require targeted chemotherapy and treatment strategies guided by accurate identification of the primary lesion. Therefore, precise diagnosis relies on integrating IHC of pleural fluid cell blocks with chest and abdominal CT imaging.

### 4.1 Cellular IHC Can Assist in the Qualitative Analysis of PE

This study demonstrated that nine cases of benign PE were diagnosed by cellular IHC, all of which were identified as mesothelial cells. A total of 14 cases of malignant PE were included in this study: 8 originated from the digestive tract, 5 from the breast, and 1 from the gynecological tract.

The selection of the appropriate antibody depends on the patient's history of primary malignancy. When malignant PE is suspected, TTF-1 and Napsin A antibodies are routinely employed [29]. Conversely, if metastatic adenocarcinoma of digestive origin is suspected, the use of CDX2, CK20, CK7 [39], and Villin is indicated. If breast origin is suspected, ER, PR [40], GATA3, and GCDFP15 should be included [31]. Similarly, if ovarian, fallopian tube, or uterine origin is suspected, Pax8 [41], Vimentin (Vim), WT1, and CA125 are recommended [41].

In patients with a history of malignancy, PE presentation can be categorized into two distinct scenarios. On the one hand, the PE may result from metastasis of the primary tumor. On the other hand, there may be no metastasis, and the PE may represent a benign effusion or inflammatory exudate. Therefore, even if a patient with a history of malignant tumors develops PE, it does not necessarily indicate metastasis. The presence of metastasis ultimately depends on the cytological identification of malignant cells in the PE.

For example, in this study, six of the nine patients with benign PE (6/9) had a history of malignant tumors. Nevertheless, IHC revealed that all pleural fluid cells were mesothelial cells, confirming benign pleural fluid.

In addition, for patients with a history of malignant tumors and suspected malignant PE, the cellular origin can be elucidated by applying IHC markers to the primary tumor tissue. For patients without a history of malignant tumors who present with PE, a panel of multiple antibody tests is required to identify the source of the effusion.

### 4.2 Imaging Examinations can Assist in Locating the Primary Tumor of PE

Imaging examinations can determine the PE location and extent. In addition, these modalities can also identify the location, range, distribution, diameter, and other metastatic characteristics of malignant tumors.

#### 4.2.1 Assessment of Disease Progression in Patients with a History of Malignant Tumors

In some cases, patients with a history of previous malignant tumors present with PE due to metastasis, and CT imaging is used to assess disease progression.

For example, a 52-year-old female patient underwent modified radical surgery for left breast cancer at an external hospital 14 years ago. The postoperative pathological diagnosis was invasive ductal carcinoma. Five years ago, she developed nodular thickening of the chest wall skin at the surgical site, suggestive of tumor recurrence, along with multiple liver metastases and sternal bone metastases, prompting the initiation of radiotherapy and chemotherapy. The patient subsequently developed PE and was admitted for pleural drainage. The diagnosis was established using fully automated IHC staining on embedded pleural effusion samples. Adenocarcinoma cells were identified; combined with the IHC results, these findings were consistent with a breast origin.

The IHC profile was as follows: HBME-1 (-), Ep-CAM (+), WT-1 (-), calretinin (CR) (-), TTF-1 (-), Napsin A (-), GCDFP15 (+), mammaglobin (-), GATA-3 (+), C-erbB-2 (2+), ER (<1%+), PR (0), CK7 (+), P40 (-).

CT imaging revealed multiple bone metastases, multiple liver metastases, two lung metastases, and bilateral PE (Fig. **3**).

Therefore, for the evaluation of PE, a full-body CT scan, including contrast-enhanced CT of the head, chest, and abdomen, is usually required. The goals are to assess the extent of disease, determine the presence of metastasis in other organs, and assist in tumor staging.

#### 4.2.2 CT and New Tumor Metastasis in PE May Indicate a New Primary Lesion or Uncover Another Hidden Primary Tumor

For example, a 84-year-old female patient underwent radical surgery for rectal cancer at an external hospital 10 years ago. One month prior to admission, she developed chest tightness and a persistent dry cough without an obvious cause. Examination at the local hospital revealed left-sided PE, and a small number of highly atypical cells were found on pleural smear, suggesting malignancy. Cell block preparation and further IHC analysis were recommended, leading to her admission to our institution.

Laboratory results showed elevated tumor markers: carbohydrate antigen CA19-9 > 1000.00 U/mL and carcinoembryonic antigen (CEA) 22.02 ng/mL.

IHC of the pleural fluid cell blocks revealed the following profile: EpCAM (+), WT-1 (-), calretinin (CR) (-), CK20 (+), CK19 (+), CDX-2 (positive in most cells), Napsin A (-), CK7 (+), TTF-1 (-), SATB2 (-), P40 (-).

The pathologists rendered a diagnosis of adenocarcinoma based on pleural fluid and cell block analysis; however, the IHC phenotype did not support a pulmonary origin. Co-expression of CK7 and CK20 in the tumor cells prompted further clinical evaluation and imaging of the pancreas, bile duct, and upper gastrointestinal tract.

Subsequent contrast-enhanced CT of the head, chest, and abdomen revealed multiple left lung lesions and irregular thickening of the left and interlobar pleura, suggestive of metastatic disease. A lesion in the tail of the pancreas was identified and suspected to be a malignant tumor (Fig. **4**).

##### 4.2.2.1 Analysis

Despite the patient's history of rectal cancer surgery, the current aetiology was neither recurrence nor metastasis of rectal cancer but rather a new primary tumor: pancreatic cancer. While cellular IHC identified the origin of the PE, imaging was indispensable for precise localisation to the pancreas.

##### 4.2.2.2 Theoretical Explanation

IHC of cells in PE cell blocks can sometimes provide only a broad indication of tissue origin (*e.g*., gastrointestinal origin). However, the digestive system encompasses many organs, including the gastrointestinal tract, liver, gallbladder, and pancreas.

#### 4.2.3 When CT Results are Unclear, or Financial Resources Allow, Whole-Body PET-CT Imaging can be Performed

For instance, a 74-year-old female patient presented with abdominal pain lasting one week, more than eight months after undergoing thyroidectomy. Serological tests revealed elevated tumor marker levels: CA125 at 189.70 U/mL, CA19-9 > 1000.0 U/mL, CA-50 > 500.0 U/mL, and CEA at 41.95 ng/mL. Gastrointestinal endoscopy showed no significant abnormalities, and adenocarcinoma cells were identified in the paraffin-embedded cell block preparation.

The IHC results were as follows: EpCAM (+), CK20 (+), CDX-2 (+), Villin (+), SATB2 (-), Napsin A (-), TTF-1 (-), Pax-8 (-), IMP-3 (-).

Prior to whole-body PET-CT imaging, the IHC marker results were analysed, and a digestive system origin was initially considered.

PET-CT revealed scattered nodular and patchy slightly high-density shadows in both lungs, with thickened and blurred lung texture; larger nodules exhibited mildly increased FDG uptake. These findings suggested a high probability of bilateral pulmonary metastasis accompanied by cancerous lymphangitis. Thickening of the gallbladder wall with local nodular increased FDG uptake raised the possibility of gallbladder cancer. Bilateral PE was detected (Fig. **5**). Additionally, in the post-thyroidectomy setting, no increased FDG uptake was observed in the surgical bed.

##### 4.2.3.1 Analysis

Although the patient had a history of thyroid cancer surgery, the current PE did not originate from thyroid cancer. IHC suggested a possible digestive system origin, and subsequent PET-CT showed no increased FDG uptake in the thyroid surgical bed. Gallbladder wall thickening with local nodular increased FDG uptake suggested the possibility of gallbladder cancer.

Malignant tumor cells exhibit an increased rate of glycolysis, a characteristic reflected by strong ^18^F-FDG uptake on PET/CT. PET/CT provides whole-body imaging, enabling simultaneous localisation of primary and metastatic lesions, thereby demonstrating high sensitivity in various malignant tumors [42]. Therefore, PET/CT has significant value in locating the origin of PE.

### 4.3 Comparison with other Works

A retrospective analysis by Xu Yi *et al*., based on the newly proposed international system for PE specimens, demonstrated that adequate serous samples, IHC, and consideration of past medical history can improve the accuracy of cytological diagnosis [43].

Similarly, Zhu *et al*. studied the diagnostic value of liquid-based cytology and cell blocks for PE and reported high diagnostic sensitivity and specificity. Overall, 96.6% of serous effusions received a directed diagnosis, including “negative for malignancy”, “malignancy”, and “suspicious for malignancy” [44].

Serous effusion cytology has high specificity and positive predictive value but modest sensitivity and negative predictive value in confirming the diagnosis of malignancy [45]. A multicentre study of over 6,000 cases also demonstrated that serous fluid cytopathology is valuable for clinical management and for developing a comprehensive understanding of benign and malignant aetiologies [46].

However, these studies did not integrate imaging into the investigation of PE [44-46].

The main causes of the high false-negative rates of effusion cytology may include surface tension, improper collection, lack of Diff-Quik-stained smears, or lack of cell blocks [47].

Several studies have reported cases using a combination of imaging and cytology for the diagnosis of PE [48, 49]. For instance, a CT scan revealed a large complex cystic lesion and PE, and subsequent cell morphology and IHC results confirmed a thyroid primary [49].

Liang *et al*. reported a case of spontaneous esophageal rupture [50]. CT findings revealed PE, pneumothorax, and mediastinal emphysema. However, the PE was not aspirated, and no cytological diagnosis was performed [50].

Olesen *et al*. investigated the relationship between PE and acute heart failure using imaging methods, including CT, to assess diagnostic accuracy [51]. However, they did not perform cytological or IHC studies on the PE [51].

Porcel studied PE in ovarian cancer patients [52]. CT was used to evaluate PE, and cytological and cell block diagnostic results were available for a subset of cases. The study concluded that ovarian cancer patients with concurrent PE have a poorer prognosis, even in the presence of small-volume effusions [52] (Table **5**).

Some of the aforementioned studies used single-modality approaches, including imaging-only [50, 51] and IHC-only [43, 45, 46] studies. In contrast, our study integrates IHC with imaging, setting it apart from these prior works.

### 4.4 Clinical Significance

Our results confirm that IHC examination accurately identifies the origin of malignant cells in all cases, while imaging studies are crucial for precise anatomical localization, particularly when the origin is non-specific (*e.g*., “digestive tract”). In benign cases, IHC detects no malignant cells; when combined with negative or inflammatory imaging findings, malignancy can be excluded. These observations support the conclusion that the combined application reduces diagnostic uncertainty.

IHC combined with imaging can guide clinical treatment decisions. If IHC identifies atypical cells as proliferating mesothelial cells and imaging suggests pulmonary inflammation or PE, the PE is diagnosed as benign.

Management strategies for benign PE include anti-inflammatory therapy, fluid and albumin supplementation, nutritional support, and maintenance of electrolyte balance. For patients with a large volume of PE, thoracentesis [53] or placement of an indwelling pleural catheter [54] may be performed for drainage.

If cell IHC identifies atypical cells as malignant, it may indicate their origin (*e.g*., the digestive system, breast, or gynecological tract). Imaging may reveal PE, lung metastasis, or other tumor manifestations. Based on these combined findings, the PE is considered malignant.

For symptomatic malignant PE, prompt local treatment is recommended. Therapeutic thoracentesis is the initial treatment measure [55]. After catheter drainage, intrapleural instillation therapy with agents such as platinum drugs [56], recombinant human endostatin [57, 58], or bevacizumab [59] is administered to inhibit malignant PE formation.

Bevacizumab is a humanized monoclonal antibody targeting VEGF that inhibits angiogenesis [60] and delays malignant PE formation.

In addition, malignant PE can also be treated with biologics and immunotherapy.

Common biological agents include IL-2 [61], tumor necrosis factor, interferon (IFN)-β [62], and IFN-γ.

For malignant PE secondary to different primary tumors, systemic anti-tumor treatment targeting the primary tumor is recommended in patients with adequate performance status.

### 4.5 Shortcomings and Limitations

#### 4.5.1 Undiagnosed PE in Patients Without a History of Malignant Tumors

The first shortcoming of this study is that, for individual patients without a history of malignant tumors, both cellular IHC and imaging may yield negative results, precluding a diagnosis of malignant PE. Consequently, the specific aetiology of the PE remains undiagnosed, necessitating patient transfer to another facility for further investigation into the underlying cause.

For example, a 72-year-old female patient presented with chest tightness and dyspnoea of unknown aetiology one month prior, with symptoms exacerbating upon exertion. Upon admission, serological testing showed no abnormalities, and IHC revealed a sparse population of atypical cells consistent with proliferating mesothelial cells. CT did not reveal any obvious primary lesion, and further PET-CT examination was performed; however, it demonstrated no significant abnormal uptake (Fig. **6**).

After a comprehensive review of the above results, the oncologist concluded that the patient's PE was not malignant but could not rule out the possibility that it was caused by conditions such as tuberculous pleurisy or connective tissue disease. To elucidate the aetiology, the patient was advised to pursue further diagnosis and treatment at another specialized hospital.

#### 4.5.2 Limited Sample Size

A second limitation is the small number of difficult cases, which precluded statistical analysis. Future studies will collect additional data to enable statistical analysis.

## CONCLUSION

In a study of 23 complex cases, the combined application of cell block IHC and imaging achieved 100% diagnostic accuracy. IHC demonstrated 100% sensitivity and specificity in detecting malignant tumors and identifying their origin, while imaging successfully localized the primary tumor or metastasis in all malignant cases.

A single diagnostic method is insufficient to address the two major challenges: tracing non-pulmonary malignant PE and distinguishing benign PE in patients with a history of tumors. The combination of cell block IHC and imaging offers complementary advantages, integrating “qualitative” and “localisation” approaches. Cell block IHC provides definitive differentiation between benign and malignant conditions, offers cellular-level classification, and can reveal otherwise hidden primary tumors [63]. Imaging detects suspicious lesions [64] and identifies the primary site while corroborating IHC findings. Together, these modalities significantly improve diagnostic accuracy for difficult PE cases, reducing misdiagnosis and missed diagnoses.

Deep learning models can extract lung information from CT images to predict pneumonia risk [65] and can also detect PE in patients [18, 19]. Moreover, AI models that integrate clinical parameters, such as chest CT, PET-CT, and tumor markers, facilitate the distinction between benign and malignant effusions [66].

Future research should focus on combining AI models, imaging, and immunohistochemistry of pleural fluid cells to differentiate between benign and malignant PE.

The present study aims to assist clinicians in diagnosing difficult pleural effusion cases and determining whether the effusion is benign or malignant.

In summary, the combination of cell block IHC and imaging modalities holds significant value in the differential diagnosis of benign and malignant PE, particularly for difficult cases and those of unknown aetiology.

## Figures and Tables

**Fig. (1):**
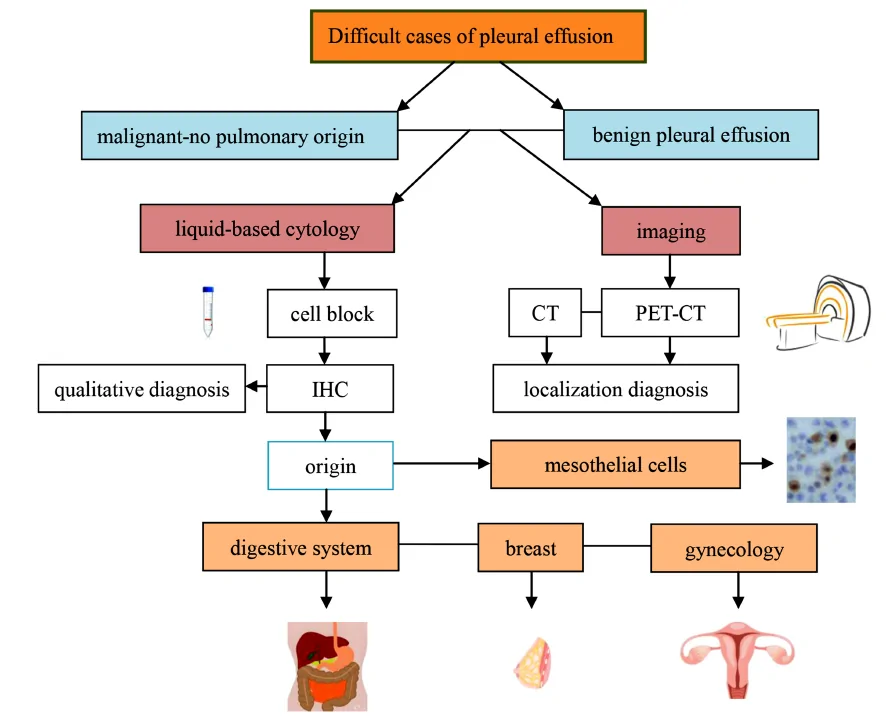
Research roadmap.

**Fig. (2):**
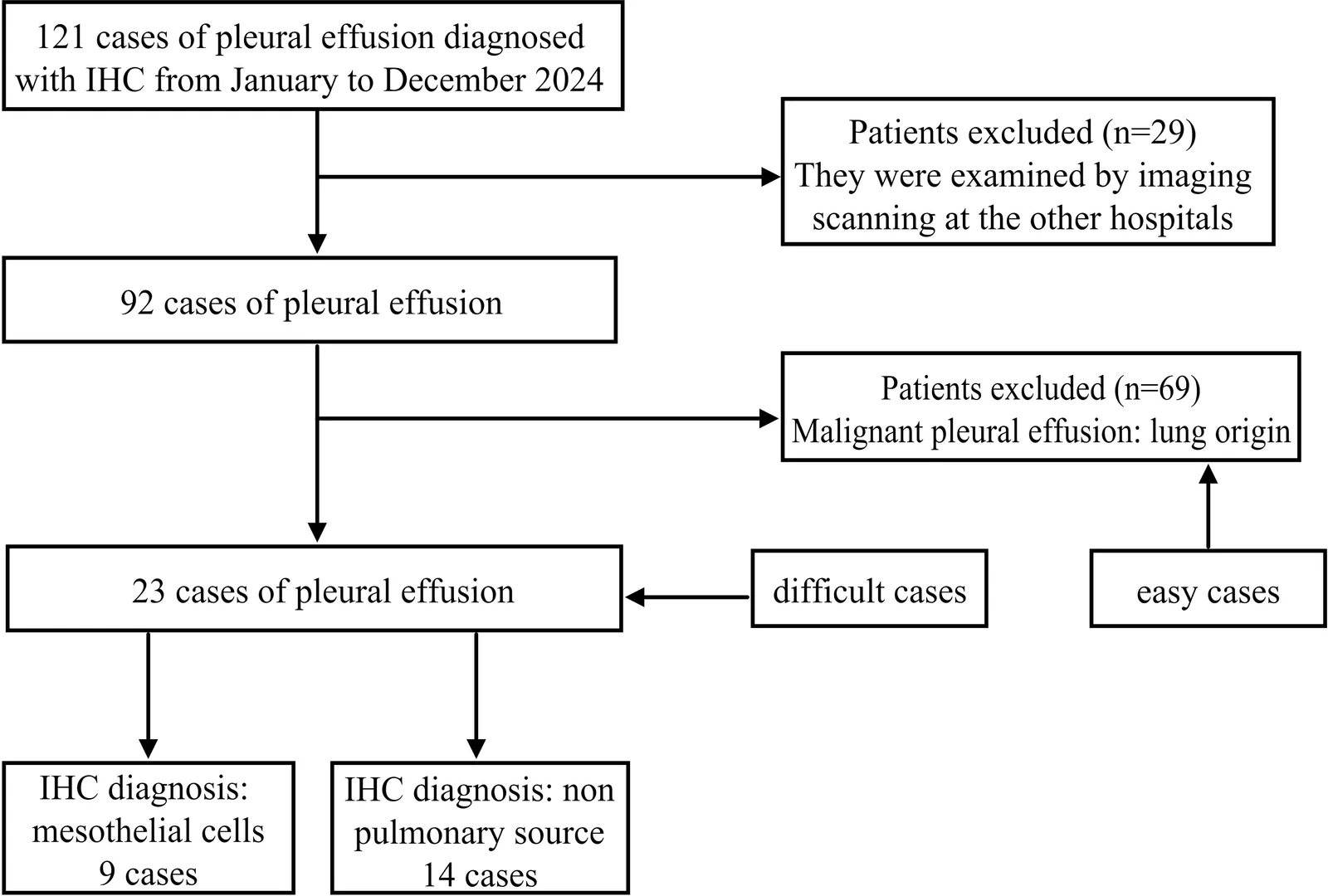
Flowchart.

**Fig. (3):**
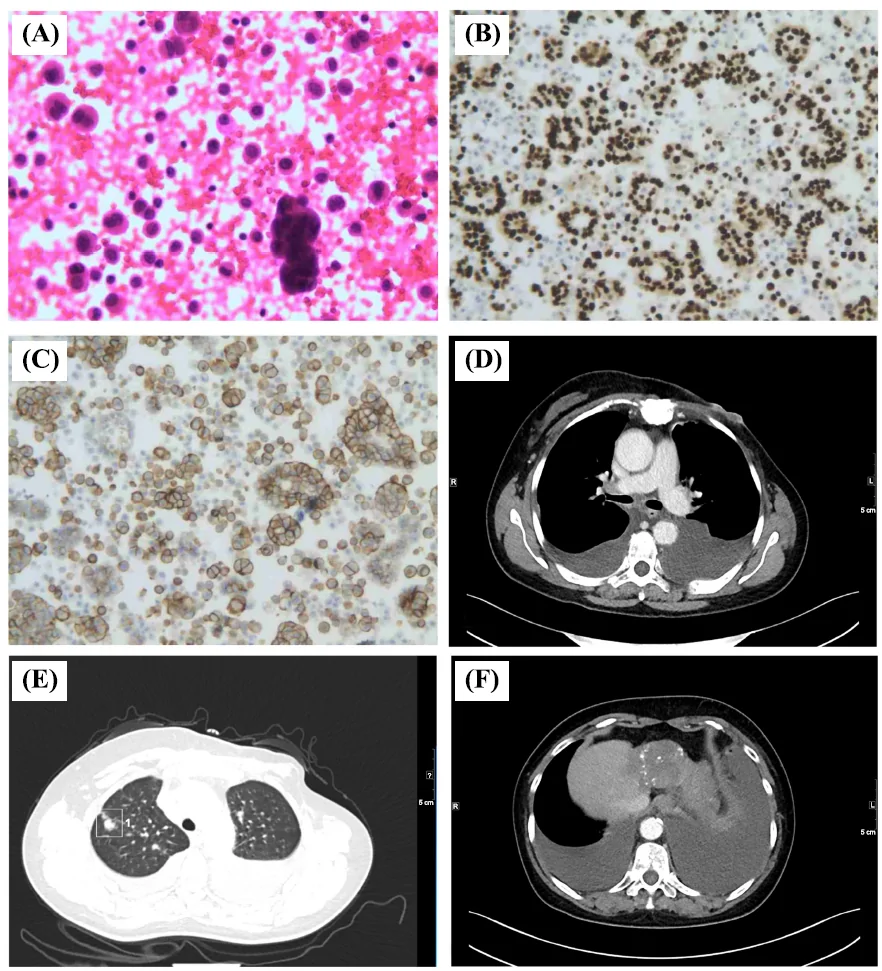
Female, 52: 14-year history of breast cancer. **A**: LCT: a few atypical cells observed (×100). **B**: GATA marker-positive (×100). **C**: C-erbb-2 marker-positive (×100). **D**: Pleural effusion. **E**: Lung metastasis. **F**: Liver metastasis.

**Fig. (4):**
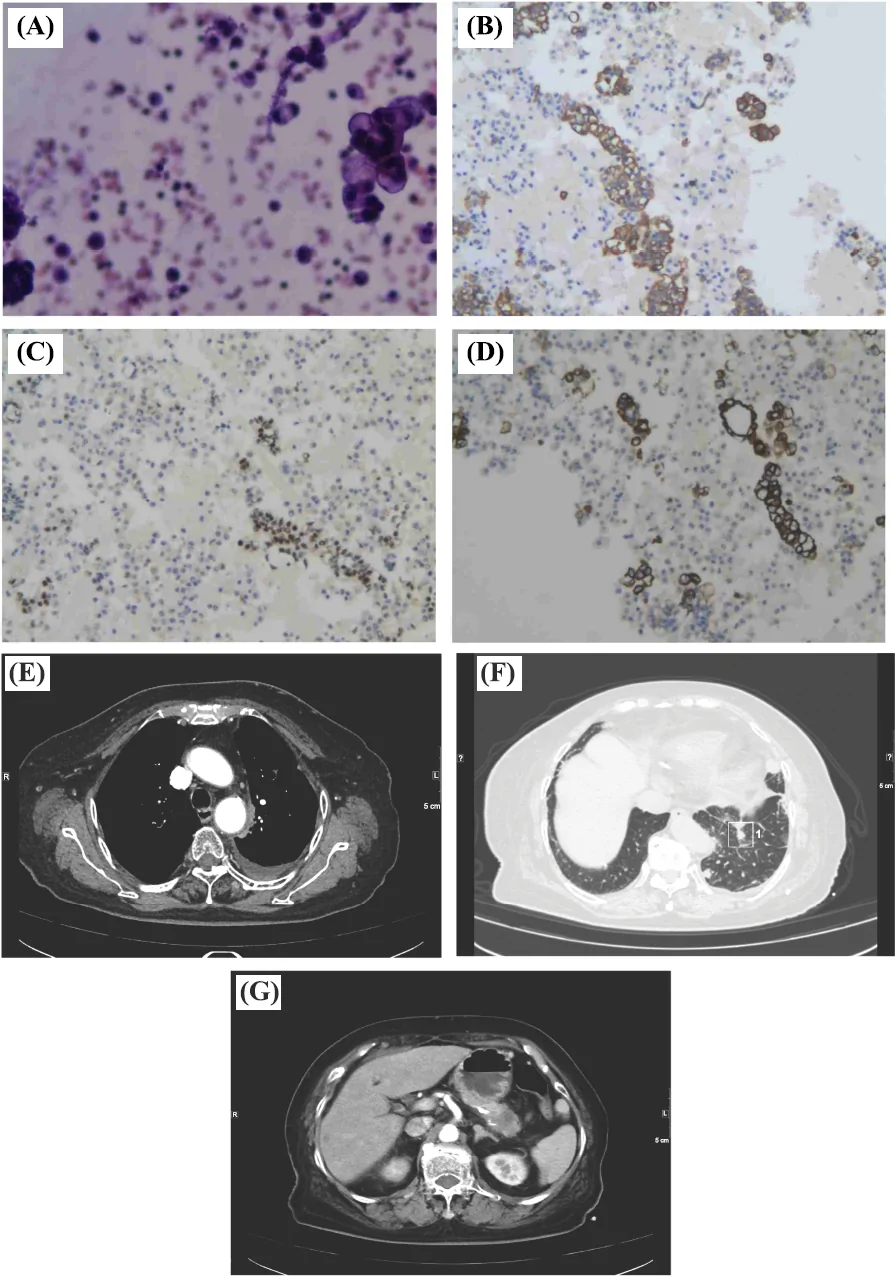
One female, 84, underwent rectal cancer surgery 10 years ago. She experienced chest tightness and irritating cough without obvious cause one month ago. **A**: A small number of highly atypical cells are observed, indicating a tendency towards cancer (×100). **B**: CK7 immunohistochemical marker-positive (×100). **C**: CDX-2 positive (×100). **D**: CK20 positive (×100). **E**: Pleural effusion. **F**: Lung metastasis. **G**: Pancreatic tail tumor.

**Fig. (5):**
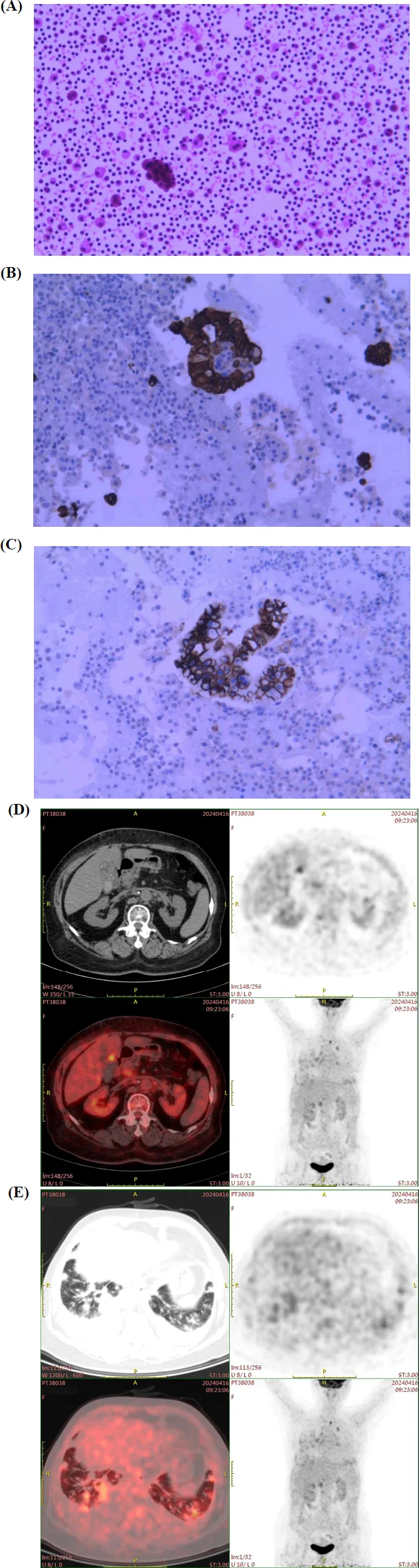
One female, 74, who had undergone thyroid cancer surgery for over 8 months, experienced abdominal pain and difficulty breathing for 1 week, IHC identified adenocarcinoma cells and, based on immunohistochemical results, first considers the source to be from the digestive system. **A**: LCT showed atypical cells, indicating a tendency towards cancer (×100). **B**: CK20 marker-positive (×100). **C**: EP-CAM marker-positive (×100). **D**: Increased FDG metabolism in the gallbladder area suggests a high possibility of gallbladder cancer. **E**: PET-CT disclosed scattered nodular and patchy slightly high-density shadows in both lungs, with increased FDG metabolism, suggesting lung metastasis.

**Fig. (6):**
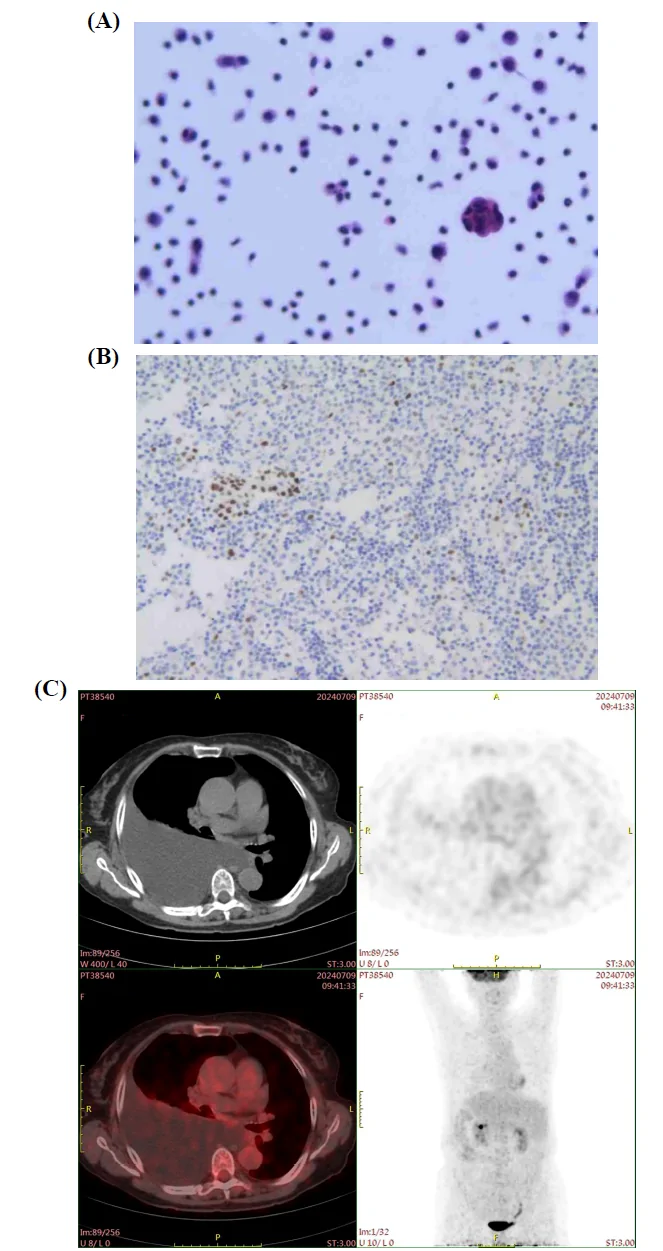
Female, 72 years old, with no history of cancer. **A**: LCT disclosed a very small amount of abnormal cells, which were observed on the pleural effusion smear (×100). **B**: IHC revealed a small amount of atypical cells that were proliferating mesothelial cells (×100). **C**: PET-CT shows no significant abnormal uptake.

**Table 1:** Flow table of case selection.

-	Cases Selection	-	-	-	Explanation
-	121 cases of PE diagnose with IHC	-	-	-	January-December in 2024
Patients excluded	29 cases	-	-	-	imaging scan in other hospitals
Remaining	92 cases	-	-	-	-
Patients excluded	69 cases	-	Easy diagnosis of PE	-	malignant PE: lung origin
Remaining	23 cases	-	Difficult diagnosis of PE	-	-
-	-	-	**benign** **9 cases**	**malignant** **14 cases**	-
-	-	-	IHC: mesothelial cells	IHC: Non pulmonary source	-
Medical history	A history of malignant tumor	Esophageal carcinoma	3cases	1 case: Intestinal source/origin	-
		gastric carcinoma	2 cases	2 cases: intestinal origin	-
		Breast carcinoma	1 case	5 cases: breast origin	-
		Cervical carcinoma	-	1 case: female reproductive tract source	-
		Thyroid carcinoma	-	1 case: digestive tract origin; PET-CT gallbladder carcinoma	Imaging: new tumor
		Colon carcinoma	-	3 cases: Intestinal source	-
		Rectal carcinoma	-	1 case: digestive tract origin; CT: pancreatic tail tumor	Imaging: new tumor
	The patient had no history of malignant tumors	-	2 cases: Anti inflammatory treatment for patients	-	-
			1 case	-	PET-CT negative, patient transferred to another hospital

**Abbreviations:** PE: pleural effusion; IHC: immunohistochemistry.

**Table 2:** Immune-positive localization markers for cancer cell blocks.

-	Mesothelial Cells	Breast Cancer	Squamous Cell Carcinoma	Ovarian Cancer	Gastrointestinal, Pancreatic, and Biliary Tract Tumors	Lung Adenocarcinoma	Colorectal Cancer
Cell membrane	HBME-1	C-erbB-2	Ep-CAM	CA125	CEA	-	-
Cytoplasm	Calretinin	GCDFP15	CK5/6	Vim	CK20,Villin, CK7	NapsinA	-
Nucleus	WT-1	ER,PR,GATA3	-	PAX-8	CDX2	TTF-1	SATB2

**Abbreviations:** HBME-1:Hector Battifora mesothelial cell-1; Ep-CAM:Epithelial cell adhesion molecule; WT-1: Wilms' tumor-1.

GCDFP15:gross cystic disease fluid protein 15; ER: Estrogen Receptor; PR: Progesterone Receptor ; GATA3: GATA binding protein 3.

PAX-8:Paired box gene 8; TTF-1:thyroid transcription factor-1; SATB2: Special AT-rich sequence-binding protein 2.

CK: Cytokeratin; CDX2: caudal type homeobox 2.

**Table 3:** The main results of LCT, IHC and imaging.

-	-	Cases	LCT	IHC	Imaging
Benign	-	9	100% (9/9) atypical cells	9/9 mesothelial cells (HBME-1+, Calretinin+, WT-1+)	CT (6 cases): 5 cases with pneumonia; PET-CT (3 cases): 2 cases of pneumonia, 1 case without significant abnormal uptake
Malignant	Digestive system origin	8	Atypical cell: 6 cases carcinoma cell: 2 cases	CK20+, CDX-2+ CK7+, TTF-1- Napsin A-	Localization: gallbladder 1 case, pancreatic tail 1case, colon 2cases, stomach 1case; abdominal metastasis/lymph node enlargement 3 cases
	Breast origin	5	Atypical cell: 3 cases carcinoma cell: 2 cases	GATA-3+, GCDFP15+, ER/PR+	Multiple site metastasis: 3 cases of lung metastasis, 2 cases of liver metastasis, and 2 cases of bone metastasis
	Gynecological origin	1	atypical cell	Pax-8+,P16+, ER/PR-	Lung metastasis

**Abbreviations:** LCT: Liquid Cytopathology Test; IHC: immunohistochemistry.

**Table 4:** The main treatment methods for benign and malignant pleural effusion (cases).

Pleural Effusion	Cisplatin Thoracic Infusion	Recombinant Human Endostatin Thoracic Perfusion	Capecitabine Chemotherapy	Intrathoracic Injection of Fluorouracil	Drainage of Pleural Effusion	Anti-infection	Oxygen Inhalation and Fluid Replacement	To another Specialized Hospital
Benign	0	0	0	0	3	7	3	1
Malignant	3	2	2	3	3	0	4	0

**Table 5:** IHC and imaging research on pleural effusion.

Author	Year	Cases	LBC/ IHC	Imaging	Results	Significance
Hawes *et al*. [49]	2025	Case report: thyroid carcinoma metastatic to pleural fluid	CK7 and PAX-8 positive; TTF-1, Napsin-A, and mesothelial markers negative	CT: complex cystic lesion on the anterior thyroid gland	CT: lung nodules and a large left pleural effusion.	Radiology, cytology, and IHC indicated thyroid origin
Liang *et al*. [50]	2025	A rare case report: esophageal rupture	-	CT	CT indicated left hydropneumothorax and mediastinal emphysema PEs: elevated CA199, CA125, and CEA levels	CT: no thoracic tumor tumor markers; concomitant changes during esophageal rupture.
Olesen *et al*. [51]	2025	Pulmonary congestion and pleural effusion	-	Chest radiography, lung ultrasound, CT	Chest radiography, a first-line diagnostic tool	CT is critical in accurately differentiating between various causes of dyspnea
Lu *et al*. [46]	2024	6177 serous fluid specimens in three hospitals	Diagnostic utility of the cell block after ISRSFC classification	-	4838 pleural effusion specimens: 92 were SFM (1.9%), and 425 were MAL (8.8%). Organ of cancer origin: Lung, liver, colon, breast, esophagus	ISRSFC is useful
Porcel *et al*. [52]	2021	189 pleural effusions (PEs) associated with ovarian cancer (OC)	Smears/cell blocks	CT	PEs on CT images	PEs: a poor prognosis
Xu *et al*. [43]	2021	2454 pleural effusion specimens were classified into ND, NFM, AUS, SFM, and MAL groups, respectively	LBC and IHC	-	Among the 36 UPS patients, the most common site of origin was the lung (36.1%), followed by the ovary (13.9%) and the breast (11.1%), *via* immunocytochemistry of cell block	ISRSFC is a standardized classification system when applied to pleural effusions.
Lobo *et al*. [45]	2021	1496 pleural effusion samples were reclassified	All pleural admitted for cytological analysis were reviewed and reclassified according to the ISRSFC	-	The ROM was, respectively, for ND, NFM, AUS, SFM, and MAL categories, with 57.1%, 23.9%, 50%, 76.2%, and 100% pleural effusions	Serous effusion cytology has a high specificity and positive predictive value

**Abbreviations:** LBC: liquid-based cytology; IHC: immunohistochemistry; ISRSFC: The International System for Reporting Serous Fluid Cytopathology; ND: nondiagnostic; NFM: negative for malignancy; AUS: atypia of uncertain significance; SFM: suspicious for malignancy; MAL: malignant; UPS: unknown primary site; ROM:Risk of malignancy; -: Not used.

## Data Availability

The imaging data, including figures and photographs associated with this paper is available on PACS(picture archiving and communication system) in the department of radiology and HIS (Hospital Information System) in Nantong Tumor Hospital, and the data can be accessed by professional staff in the department of radiology through login PACS and HIS. The pathological data were obtained from the pathological image database of Nantong Tumor Hospital, China.

## LIST OF ABBREVIATIONS

PE: Pleural Effusion
LBC: Liquid-Based Cytology
CT: Computed Tomography
PET CT: Positron Emission Tomography-Computed Tomography
IHC: Immunohistochemistry
LCT: Liquid Based Cytopathology Test
CR: Calretinin
FDG: Fluorodeoxyglucose
TTF 1: Thyroid Transcription Factor 1
CK: Cytokeratin
WT 1: Wilms Tumor Protein 1
GATA 3: GATA Binding Protein 3
GCDFP 15: Gross Cystic Disease Fluid Protein 15
CDX2: Caudal Type Homeobox 2
Ep CAM: Epithelial Cell Adhesion Molecule
CEA: Carcinoembryonic Antigen
PAX-8: Paired Box Protein 8
ER: Estrogen Receptor
PR: Progesterone Receptor
CA125: Cancer Antigen 125
CA199: Carbohydrate Antigen 19 9
Ki67: Proliferation Marker Ki 67
VEGF: Vascular Endothelial Growth Factor
IL 2: Interleukin 2
IFN: Interferon
AI: Artificial Intelligence

## STANDARDS OF REPORTING

STROBE guidelines were followed.
